# Trends in clinical research in Saudi Arabia: A registry-based descriptive analysis of ClinicalTrials.gov to 2025

**DOI:** 10.1097/MD.0000000000048740

**Published:** 2026-05-15

**Authors:** Mohammed M. Aldurdunji

**Affiliations:** a Pharmaceutical Practices Department, College of Pharmacy, Umm Al-Qura University, Makkah, Saudi Arabia.

**Keywords:** clinical trials, registration, results reporting, Saudi Arabia, transparency

## Abstract

Clinical research activity in Saudi Arabia has expanded, but systematic characterization of trials registered on ClinicalTrials.gov remains limited. A clearer understanding of portfolio composition, geographic distribution, and transparency indicators may support research planning and capacity development. To describe the characteristics and time trends of clinical studies registered on ClinicalTrials.gov with at least one Saudi site through 2025, focusing on sponsor type, study phase, geographic distribution, and results posting. A descriptive registry-based analysis was conducted of 1502 studies registered through August 2025. Studies were classified by sponsor, design, phase, country scope, enrollment, and therapeutic area. Geographic distribution was summarized at the city level. Registry-based transparency was assessed as the proportion of studies with posted results overall and among completed studies. Most studies were interventional (75.5%), with greater activity in later phases such as Phase 3 and limited early-phase research. Nonindustry sponsors led 73.0% of registrations. Planned enrollment was modest, with half of the trials targeting 100 or fewer participants. Trial activity was concentrated in Riyadh, with smaller hubs in Jeddah and Dammam. Transparency was low, with only 12.1% of studies posting results. Among completed trials, industry sponsors reported results more frequently than nonindustry sponsors. Neurology, oncology, and endocrine disorders were the most represented areas, while cardiovascular and respiratory research were comparatively underrepresented. Saudi Arabia’s clinical research portfolio shows growth in late-phase activity and increasing participation in multinational studies. The observed geographic concentration and low rates of results posting, particularly among nonindustry sponsors, suggest opportunities to strengthen early-phase infrastructure, broaden participation beyond major hubs, and support timely and consistent results disclosure through institutional processes.

## 1. Introduction

Clinical trials are a central mechanism for evaluating therapeutic efficacy and safety, informing regulatory and reimbursement decisions, and updating clinical practice.^[1]^ International guidance and legal frameworks over the past 2 decades have strengthened expectations for prospective registration, protocol completeness, and timely results reporting, contributing to broader emphasis on transparency and reproducibility.^[2–4]^ Although global trial activity has grown substantially, the pace and composition of this growth vary across regions, sponsors, and therapeutic areas, and reporting performance remains inconsistent.^[5,6]^

In the Middle East and North Africa, and particularly within the Gulf Cooperation Council (GCC), research activity has increased but remains concentrated in major academic medical centers, with limited industry leadership and operational delays that affect trial start-up and dissemination.^[7,8]^ In Saudi Arabia, health sector transformation under Vision 2030 has prioritized research capability and international collaboration, and earlier assessments have described a maturing national portfolio.^[9–11]^ Recent literature has characterized Saudi clinical trial activity using registry-based analyses and stakeholder perspectives; however, existing work often focuses on specific trial subsets or on operational challenges (e.g., infrastructure, funding, and regulatory timelines) rather than providing an updated whole-portfolio description that combines portfolio composition, city-level distribution, and registry-based transparency indicators.^[12,13]^ However, gaps persist across the broader clinical research ecosystem. Previous analyses highlight selective representation of therapeutic areas, particularly relative to cardiometabolic burden, and low rates of results disclosure among academically led studies.^[4,14,15]^ These gaps have direct implications for national research planning and alignment with disease burden. International experience shows that coordinated policy, infrastructure investment, and regulatory facilitation can accelerate both early- and late-phase capacity, offering useful benchmarks for regional development.^[16,17]^

An updated long-horizon assessment may help evaluate how Saudi Arabia’s clinical research activity has evolved and how it aligns with national priorities. ClinicalTrials.gov provides a comprehensive and standardized registry that enables consistent classification of study designs, phases, sponsors, geographic distribution, therapeutic focus, multinational participation, and results reporting, while supporting regional and international comparisons.^[18]^ Using this registry, the present study aims to describe the landscape and temporal trends of clinical studies registered on ClinicalTrials.gov with at least one Saudi site through 2025. Specifically, we aim to summarize study design, phase occurrence (interventional studies), sponsor type, planned enrollment, geographic distribution by city, country scope (Saudi-only vs multicountry), therapeutic orientation, posted-results availability (overall and among completed trials), and eligibility patterns by sex and age. These descriptive findings are intended to support research planning, transparency initiatives, and capacity development.

## 2. Methods

### 2.1. Study design and data source

This study was conducted as a cross-sectional, descriptive registry analysis of clinical studies registered on ClinicalTrials.gov with at least one study location in Saudi Arabia. All summaries reflect the registry information available at the time of data extraction.

### 2.2. Data source and cohort identification

ClinicalTrials.gov served as the sole data source. Records were retrieved on August 10, 2025 by identifying studies whose locations module listed at least one Saudi Arabia site and exporting the matching study records as CSV file for tabulation. The final analytic cohort included 1502 studies registered through August 2025. No restrictions were applied by recruitment status, sponsor type, therapeutic area, or study design. The raw ClinicalTrials.gov CSV export used for this analysis is provided in Table S1, Supplemental Digital Content.

### 2.3. Eligibility criteria

Studies were included if at least one listed facility was located in Saudi Arabia. Studies with sites exclusively outside Saudi Arabia were excluded. Phase-specific summaries were limited to interventional studies. Summaries of results posting were reported overall and, for sponsor comparisons, restricted to studies with overall status “Completed.” Records with missing values were retained, and denominators were restricted to records with non-missing values for the attribute being summarized.

### 2.4. Data extraction and variable definitions

For each eligible record, the following information was abstracted from ClinicalTrials.gov fields: study type, recruitment/overall status, sponsor information, phase (interventional studies), enrollment, start date, primary completion date, locations (countries and Saudi cities), conditions, and posted tabular results. Sponsor class was derived from lead sponsor and grouped as industry versus nonindustry. Country scope was classified as Saudi-only when all listed countries were Saudi Arabia and as multicountry when any additional country was present.

Phase assignments followed ClinicalTrials.gov terminology. Trials reporting combined phases (e.g., Phase 1/2 or Phase 2/3) were counted in each relevant phase (inclusive counting), so phase percentages do not sum to 100%. Enrollment used the numeric enrollment field, prioritizing “Actual” values when available, and was summarized as 0 to 100, 100 to 500, and >500 participants. Sex eligibility was taken from the “Sex” field, and age eligibility used ClinicalTrials.gov age bands (child, adult, and older adult); because trials may include multiple age groups, age-category counting was inclusive.

Therapeutic areas were assigned by mapping free-text condition terms to consolidated categories informed by MeSH-style groupings and national clinical practice domains; mapping was inclusive when multiple conditions were listed.

### 2.5. Geographic normalization

Facility locality was standardized to Saudi cities. Free-text facility and city strings were normalized by trimming whitespace, harmonizing common transliteration variants, removing honorifics and institutional suffixes embedded in the city field, and collapsing near-duplicate spellings. Entries that were not cities were recoded to the correct city when determinable from the facility string and were otherwise excluded from city-level tallies. For city distributions, counts reflected unique trial-city pairs, such that a trial operating in multiple cities contributed once to each city’s numerator; percentages were calculated against the cohort denominator and therefore did not sum to 100%.

### 2.6. Time indexing and partial years

Activity and phase trajectories were indexed by start year, and completion and results-posting summaries were indexed by primary completion year. Because 2025 was incomplete at the time of extraction, 2025 values were reported descriptively and labeled as partial in cumulative plots.

### 2.7. Statistical analysis and software

Analyses were descriptive. Counts and percentages were reported with denominators restricted to records with non-missing values for the attribute of interest. Inclusive counting was applied for phase and therapeutic area, allowing a record to contribute to multiple categories; percentages for these outcomes were interpreted as the share of trials that include the category rather than mutually exclusive fractions. Time-series displays used annual indexing without smoothing, and cumulative plots were constructed by summing annual counts through each index year. Comparative summaries were stratified by sponsor class and, where relevant, by country scope. All tabulations and figures were produced in Microsoft Excel (Microsoft Corporation).

### 2.8. Ethics

Only publicly available study-level registry data were used, and no identifiable information was accessed or analyzed; therefore, Institutional Review Board (IRB) approval and informed consent were not required.

## 3. Results

### 3.1. Characteristics of included trials

A total of 1502 studies were identified in ClinicalTrials.gov with at least one site in Saudi Arabia (Table 1). Most were interventional (1134, 75.5%), with 366 observational studies (24.4%) and 2 expanded access records. Among interventional trials, early-phase activity was limited, with 9 Early Phase 1 records (0.8%) and 44 Phase 1 records (3.9%). Later-phase activity was more frequent, including 127 Phase 2 studies (11.2%), 274 Phase 3 studies (24.2%), and 109 Phase 4 studies (9.6%). A substantial proportion (615, 54.2%) were classified as “Not Applicable,” consistent with nondrug interventions. Because some trials reported combined phases, phase totals reflect overlapping assignments and do not sum to a single denominator (Table 1).

**Table 1:** Descriptive characteristics of clinical trials registered in Saudi Arabia.

Characteristic	Number	Percentage (%)
Total trials	1502	
Study type – interventional	1134	75.5
Study type – observational	366	24.4
Study type – expanded access	2	0.1
Phases (inclusive; interventional denominator n = 1134)		
Early Phase 1	9	0.8
Phase 1	44	3.9
Phase 2	127	11.2
Phase 3	274	24.2
Phase 4	109	9.6
Not applicable	615	54.2
Enrollment (non-missing denominator n = 1501)		
0–100	752	50.1
100–500	431	28.7
>500	318	21.2
Sponsor class		
Sponsor class – industry	405	27
Sponsor class – nonindustry	1097	73

Planned enrollment was modest. Among trials with non-missing enrollment (n = 1501), half planned to recruit 0 to 100 participants (752, 50.1%). Medium-sized trials (100–500 participants) accounted for 431 studies (28.7%), and 318 studies (21.2%) planned more than 500 participants. Sponsorship was predominantly nonindustry, with 1097 studies (73.0%) led by academic, governmental, or nonprofit entities and 405 studies (27.0%) led by industry.

### 3.2. Trial status and results availability

Eligibility criteria were generally inclusive. Female participation was permitted in 1420 trials (94.5%) and male participation in 1371 trials (91.3%), indicating that some studies restricted eligibility to a single sex. Adults aged 18 to 64 years were eligible in 1310 trials (87.2%), older adults in 912 trials (60.7%), and children in 491 trials (32.7%).

Most studies were completed at the time of extraction (933, 62.1%). Smaller proportions were recruiting (154, 10.3%) or not recruiting (122, 8.1%), while 66 studies (4.4%) were terminated or withdrawn. Registry results were available for 181 studies (12.1%), while 1321 studies (87.9%) had no posted results. Among completed trials, industry sponsors posted results for 48% of studies, compared with 4% for nonindustry sponsors (Table 2).

**Table 2:** Eligibility, recruitment status and results availability of clinical trials registered in Saudi Arabia.

Characteristic	Number	Percentage (%)
Gender		
All sexes	1502	100.0
Female	1420	94.5
Male	1371	91.3
Participant age		
Child (birth–17)	491	32.7
Adult (18–64)	1310	87.2
Older adult (65+)	912	60.7
Status group		
Not recruiting	122	8.1
Recruiting	154	10.3
Completed	933	62.1
Terminated/withdrawn	66	4.4
Unknown	227	15.1
Results availability		
Has results	181	12.1
No results available	1321	87.9

### 3.3. Geographical and institutional distribution

Trial activity was concentrated in major research hubs. Riyadh accounted for 852 trials (56.7%), followed by Jeddah (294, 19.6%) and Dammam (134, 8.9%). Other cities with notable activity included Makkah (83, 5.5%), Al Kharj (50, 3.3%), Al Khobar (49, 3.3%), Buraidah (33, 2.2%), Jazan (30, 2.0%), Taif (28, 1.9%), and Abha (26, 1.7%). Overall, trial activity was most frequently recorded in Riyadh, followed by Jeddah and Dammam (Table 3).

**Table 3:** Distribution of included trials across Saudi cities: top 10 (per-trial presence; normalized city names).

City	Trials	Percentage (%)
Riyadh	852	56.7
Jeddah	294	19.6
Dammam	134	8.9
Makkah	83	5.5
Al Kharj	50	3.3
Al Khobar	49	3.3
Buraidah	33	2.2
Jazan	30	2.0
Taif	28	1.9
Abha	26	1.7

Counts reflect unique trial–city pairs, such that trials operating in multiple cities contribute once to each city’s total; percentages use the cohort denominator and therefore do not sum to 100%.

### 3.4. Clinical focus

Therapeutic-area patterns are presented in Table 4. Neurology was the most represented field (279 trials, 18.6%), followed by oncology (238, 15.8%), endocrine and metabolic disorders (207, 13.8%), infectious diseases (182, 12.1%), and musculoskeletal and rheumatology (168, 11.2%). Cardiovascular (91, 6.1%) and respiratory disease (27, 1.8%) were comparatively underrepresented. A substantial proportion of studies (473, 31.5%) did not map to predefined categories. Because trials may list multiple conditions, therapeutic-area percentages are not mutually exclusive.

**Table 4:** Distribution of clinical trials by therapeutic area in Saudi Arabia.

Therapeutic area	Number	Percentage (%)
Neurology	279	18.6
Oncology	238	15.8
Endocrine & metabolic	207	13.8
Infectious diseases	182	12.1
Musculoskeletal & rheumatology	168	11.2
Cardiovascular	91	6.1
Gastroenterology & hepatology	88	5.9
Renal & urology	30	2.0
Respiratory	27	1.8
Hematology	26	1.7
Other/unspecified (no mapped area)	473	31.5

### 3.5. Trends and comparison of trial phase distribution by sponsor class

**Figure 1.**
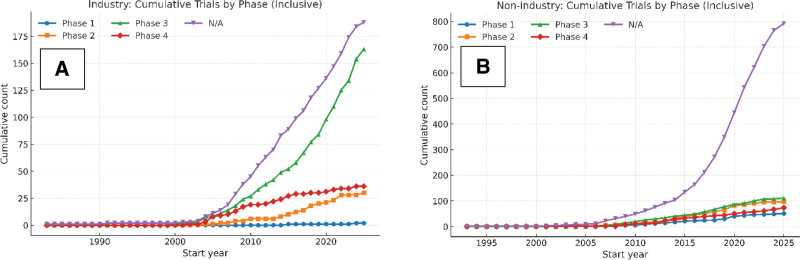
Cumulative registered trials by sponsor type and phase. These panels show cumulative trial counts by start year and phase, with Panel A for industry-sponsored trials and Panel B for nonindustry trials. “N/A” refers to studies without FDA-defined phases (e.g., behavioral, device, or procedural trials). Axes and scales are aligned for direct comparison. The distribution of “N/A” trials differs by sponsor class and is shown for descriptive comparison. FDA = Food and Drug Administration.

Industry-sponsored registrations showed an increase on late-phase development by 2025, with Phase 3 accounting for the largest number of entries (163), followed by Phase 4 (36), Phase 2 (30), and a small number of Phase 1 studies (2). A notable volume of industry-led trials (188) did not list a defined drug-development phase. These observed patterns reflected longitudinally in Figure 1.

**Figure 2.**
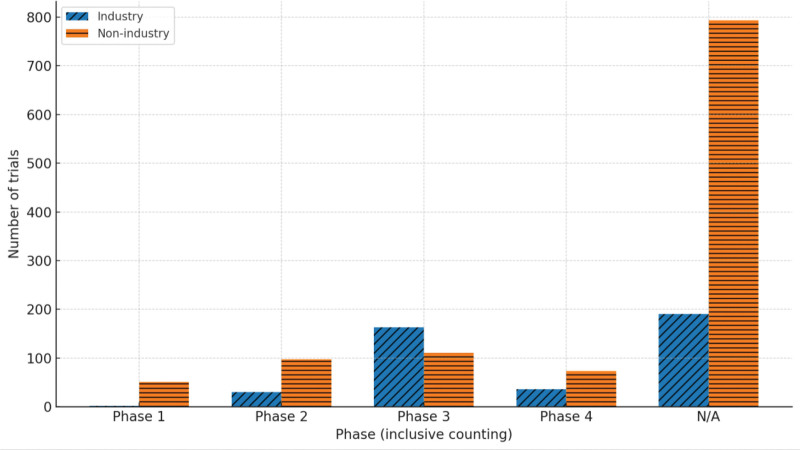
Clinical trial phase distribution by sponsor type. This bar chart illustrates the distribution of clinical trials across development phases (Phases 1–4, plus an unspecified “N/A” category), segmented by sponsor type: industry and nonindustry. The nonindustry sponsors account for a large proportion of trials with unspecified phases, while industry sponsors are more heavily represented in Phase 3. This visualization summarizes phase distributions by sponsor category.

Nonindustry sponsors displayed a different configuration, dominated by studies classified as “Not Applicable,” which reached 792 registrations by 2025. Their activity in Phases 1 to 4 remained comparatively limited (51, 97, 111, and 73, respectively), with many studies involving devices, procedures, and behavioral interventions more than conventional drug-development phases (Fig. 2).

### 3.6. Trends and comparison of trial completion and results reporting

**Figure 3.**
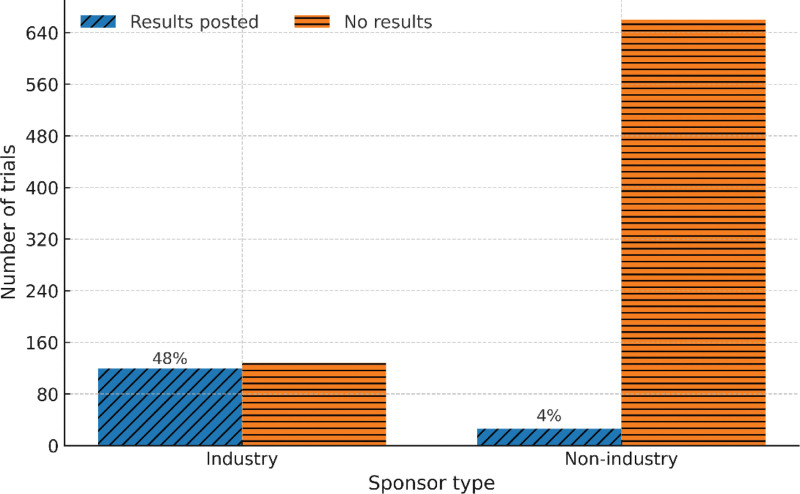
Reporting of results in completed trials by sponsor type. This bar chart shows completed clinical trials in Saudi Arabia by sponsor type and whether results were posted on ClinicalTrials.gov. Results were posted for 48% of industry-sponsored trials, but only 4% of nonindustry-sponsored trials, showing differences in posted-results availability by sponsor type.

**Figure 4.**
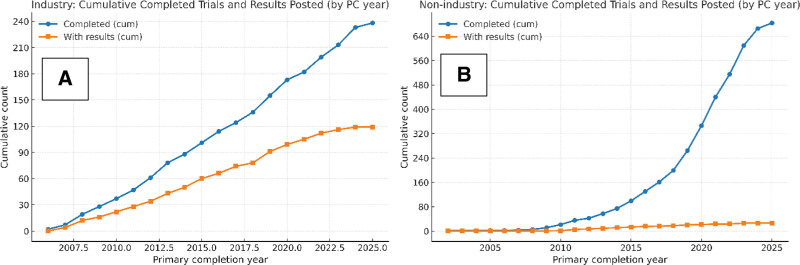
Cumulative completed trials and results reporting by sponsor type (by year). Panels show the cumulative number of completed clinical trials and those with posted results on ClinicalTrials.gov by primary completion year, stratified by sponsor type (A: Industry, B: Nonindustry). Panels display cumulative counts of completed trials and those with posted results by sponsor type over time.

Among completed studies, results were posted for 48% of industry-led trials (119/247) compared with 4% of nonindustry trials (26/686; Fig. 3). Figure 4 presents cumulative counts of completed trials and cumulative counts of trials with posted results by primary completion year, stratified by sponsor class. In the nonindustry group, cumulative completions increased steadily after 2015, while cumulative posted results increased at a slower rate over the same period. In the industry group, cumulative posted results increased more closely in parallel with cumulative completions across years. Across both sponsor classes, the cumulative number of trials with posted results remained substantially lower than the cumulative number of completed trials.

## 4. Discussion

This registry-based analysis shows substantial growth in Saudi Arabia’s clinical research activity, characterized by a predominance of interventional designs and a strong Phase III presence. Trial conduct remains highly concentrated in Riyadh, with secondary hubs in Jeddah and Dammam. Despite overall expansion, key national needs, particularly cardiovascular and respiratory research, continue to be underrepresented, and results reporting remains limited, especially among nonindustry sponsors. The present analysis strengthens these earlier observations by confirming that these structural patterns have persisted over time, consistent with prior national audits.^[9,10]^ It also provides new contributions by extending the evaluation through 2025, which allows clearer identification of emerging trends, including shifts in sponsor-specific phase activity and the growing divergence between trial completion and public results reporting, an issue repeatedly highlighted in international assessments of transparency.^[4]^ Together with the alignment of findings with national clinical priorities^[14]^ and earlier discussions of reporting and operational challenges,^[19,20]^ these additions offer a more detailed understanding of how the national clinical research portfolio is evolving and highlight elements that were not captured in earlier evaluations.

Saudi Arabia’s trajectory parallels patterns observed across the GCC, where research is predominantly driven by academic centers and industry involvement remains comparatively modest.^[8,10]^ In contrast, markets such as China have rapidly expanded both early- and late-phase capacity through coordinated regulatory reforms and infrastructure investment.^[16]^ Similarly, the United States and European Union demonstrate more balanced portfolios but continue to face persistent disclosure challenges.^[1,4]^ These international models illustrate how policy alignment and streamlined oversight may accelerate scale and participation in global development pipelines.

The limited number of early-phase studies highlights constrained translational capacity, consistent with regional analyses reporting underdeveloped Phase I/II infrastructure.^[21]^ Strengthening early-phase activity may require specialized pharmacology units, round-the-clock clinical monitoring capabilities, and reliance-based ethics pathways that reduce start-up timelines.^[22,23]^ Given that trial execution relies on sustained recruitment, the current geographic centralization presents operational challenges: concentration in Riyadh may limit equitable access for peripheral regions and constrain enrollment for high-burden conditions. International evidence suggests that expanding trial networks to community and secondary hospitals improves representativeness and accrual,^[24]^ while inspections and global operations literature continue to highlight the delays introduced by fragmented contracting and multilayered ethics review.^[20,25]^

Sponsor dynamics further shape the national portfolio. Nonindustry institutions remain the dominant drivers of trial activity, whereas industry sponsors contribute mainly to late-phase drug development. Although academic leadership supports locally relevant research, limited sponsor diversity may hinder integration into multinational programs.^[10]^ Comparative models from countries that expanded rapidly through public–private partnerships and streamlined governance structures suggest feasible approaches to broaden engagement.^[17,26]^

Transparency remains a major gap. Only a small fraction of completed trials posted results, with a notable disparity between industry and nonindustry sponsors. This mirrors global patterns in which academic institutions exhibit lower compliance with results disclosure requirements.^[5]^ Non-reporting diminishes scientific value and undermines public trust. International regulatory experience indicates that enforceable timelines, institutional accountability mechanisms, and integration of reporting expectations into IRB and funding workflows can substantially improve compliance.^[2,3,20]^

The therapeutic-area distribution shows strong representation in neurology, oncology, and endocrine disorders, contrasted with lower activity in cardiovascular and respiratory fields. This imbalance is consistent with regional audits^[9]^ and sits at odds with national disease burden estimates.^[14,15]^ Rebalancing could be advanced through pragmatic trial designs, targeted funding initiatives, and co-sponsored programs that incentivize research in high-burden conditions.^[18,27]^ Although older-adult eligibility was common, international evidence consistently shows persistent under-representation in actual enrollment, underscoring the need for geriatric-appropriate recruitment and outcome strategies.^[28–30]^

Saudi Arabia increasingly participates in multinational trials, particularly in oncology and infectious diseases, though national coordination remains limited. Evidence from the collaborative coronavirus disease 2019 convalescent plasma trial demonstrates the feasibility of structured multicenter efforts,^[31]^ while comparable cardiovascular initiatives reflect growing capability.^[14]^ Advances will require GCC-wide harmonization in ethics reliance, monitoring standards, and data governance, similar to the frameworks used by established international trial networks.^[32,33]^

Regulatory processes continue to evolve, with the Saudi Food and Drug Authority advancing harmonization and oversight; however, ethics delays, variable site accreditation, and data-governanceconstraints remain important operational barriers.^[20]^ International precedents, including reliance or centralized IRBs, unified national contracts, and consolidated submission portals, have demonstrated substantial reductions in cycle time without compromising participant protection.^[22,34]^ Aligning institutional processes with Standard Protocol Items: Recommendations for Interventional Trials guidelines and good clinical practice, while linking approvals to enforceable reporting expectations, would strengthen the foundation for national capacity building.^[1–3]^

Registry-based descriptive patterns suggest 4 recurring constraints: limited early-phase capacity, geographic concentration of activity, predominance of nonindustry sponsorship, and low rates of posted results. Potential strategies could include expanding accreditation and support for early-phase units; strengthening site readiness beyond major hubs through shared Standard operating procedures and reliance-based review pathways; encouraging sponsor diversification through co-funding mechanisms and more standardized contracting; and institutionalizing transparency through prospective registration checks and time-bound results posting expectations aligned with funder and institutional workflows.^[35]^ Examples of Saudi multicenter initiatives illustrate that coordinated research delivery is feasible when operational barriers are addressed.^[31,36]^

This study has limitations inherent to registry-based analyses. It relies on a single registry (ClinicalTrials.gov), which may not capture all trials conducted in Saudi Arabia, and registry fields may be incomplete or inconsistently updated. Results posting is subject to timing-related effects, including reporting lag, which may affect apparent results availability for more recent completion years. In addition, mapping free-text condition terms to consolidated therapeutic categories may introduce misclassification. Furthermore, as a descriptive registry analysis, this study does not include inferential statistical comparisons, and observed differences should be interpreted as descriptive patterns rather than causal effects.

## 5. Conclusions

Saudi Arabia’s clinical research portfolio has expanded in scale and complexity, with notable strength in Phase 3 activity, academic leadership, and growing participation in multinational studies. However, descriptive registry patterns suggest persistent gaps, including limited early-phase activity, geographic concentration in major hubs, predominance of nonindustry sponsorship, and low rates of posted results, particularly among nonindustry investigators. These findings highlight opportunities to strengthen early-phase infrastructure, broaden trial capacity beyond major cities, streamline start-up processes (e.g., ethics and contracting), and reinforce institutional expectations for timely results disclosure. Addressing these areas may help align future trial activity with national health priorities and support continued regional and international engagement.

## Author contributions

**Conceptualization:** Mohammed M. Aldurdunji.

**Data curation:** Mohammed M. Aldurdunji.

**Formal analysis:** Mohammed M. Aldurdunji.

**Funding acquisition:** Mohammed M. Aldurdunji.

**Investigation:** Mohammed M. Aldurdunji.

**Methodology:** Mohammed M. Aldurdunji.

**Project administration:** Mohammed M. Aldurdunji.

**Resources:** Mohammed M. Aldurdunji.

**Software:** Mohammed M. Aldurdunji.

**Supervision:** Mohammed M. Aldurdunji.

**Validation:** Mohammed M. Aldurdunji.

**Visualization:** Mohammed M. Aldurdunji.

**Writing – original draft:** Mohammed M. Aldurdunji.

**Writing – review & editing:** Mohammed M. Aldurdunji.


